# Lipidomic profiling of patient-specific iPSC-derived hepatocyte-like cells

**DOI:** 10.1242/dmm.030841

**Published:** 2017-09-01

**Authors:** Mostafa Kiamehr, Leena E. Viiri, Terhi Vihervaara, Kaisa M. Koistinen, Mika Hilvo, Kim Ekroos, Reijo Käkelä, Katriina Aalto-Setälä

**Affiliations:** 1 Faculty of Medicine and Life Sciences, University of Tampere, Tampere, 33520, Finland; 2 Zora Biosciences, Espoo, 02150, Finland; 3 Department of Biosciences, University of Helsinki, Helsinki, 00014, Finland; 4 Heart Hospital, Tampere University Hospital, Tampere, 33520, Finland

**Keywords:** Induced pluripotent stem cell, iPSC, Hepatocyte-like cell, HLC, Differentiation, Cell model, Lipidomics, Fatty acid

## Abstract

Hepatocyte-like cells (HLCs) differentiated from human induced pluripotent stem cells (iPSCs) offer an alternative model to primary human hepatocytes to study lipid aberrations. However, the detailed lipid profile of HLCs is yet unknown. In the current study, functional HLCs were differentiated from iPSCs generated from dermal fibroblasts of three individuals by a three-step protocol through the definitive endoderm (DE) stage. In parallel, detailed lipidomic analyses as well as gene expression profiling of a set of lipid-metabolism-related genes were performed during the entire differentiation process from iPSCs to HLCs. Additionally, fatty acid (FA) composition of the cell culture media at different stages was determined. Our results show that major alterations in the molecular species of lipids occurring during DE and early hepatic differentiation stages mainly mirror the quality and quantity of the FAs supplied in culture medium at each stage. Polyunsaturated phospholipids and sphingolipids with a very long FA were produced in the cells at a later stage of differentiation. This work uncovers the previously unknown lipid composition of iPSC-HLCs and its alterations during the differentiation in conjunction with the expression of key lipid-associated genes. Together with biochemical, functional and gene expression measurements, the lipidomic analyses allowed us to improve our understanding of the concerted influence of the exogenous metabolite supply and cellular biosynthesis essential for iPSC-HLC differentiation and function. Importantly, the study describes in detail a cell model that can be applied in exploring, for example, the lipid metabolism involved in the development of fatty liver disease or atherosclerosis.

## Abstract (precis)

**Summary:** The lipidome of stem cells is remodelled in response to supplies available in the cell culture media, and as a result of changing expression of genes involved in lipid metabolism, as the cells differentiate and mature towards functional HLCs.

## INTRODUCTION

The liver is the main metabolic and synthetic organ in the human body, carrying out more than 500 different functions. It is mainly composed of hepatocytes, which constitute approximately 60% of the cells in the liver and possess many important functions. Hepatocytes produce the majority of circulating plasma proteins, including transporters (such as albumin and lipoproteins), protease inhibitors (α1-antitrypsin, antithrombin and α2-macroglobulin), blood coagulation factors, and modulators of immune complexes and inflammation (complement C3, C-reactive protein). Hepatocytes also control the homeostasis of energy/fuel molecules such as glucose/glycogen and fatty acids (FAs) as well as other essential compounds of lipid metabolism such as cholesterol and bile acids. Additionally, liver has a central role in lipid metabolism as it is the major site for the generation of plasma lipoproteins (Godoy et al., 2013).

The use of hepatocytes as *in vitro* models to explore different aspects of liver function and metabolism has escalated in recent years but primary human hepatocytes (PHHs), the key *in vitro* cell type involved in e.g. cholesterol metabolism, are scarce because they are obtained from organ donors. Furthermore, when in culture the PHHs quickly dedifferentiate and lose their liver functions, thus making them impractical for modelling the liver *in vivo* (Elaut et al., 2006). Human induced pluripotent stem cell (iPSC)-derived hepatocytes provide a good alternative to PHHs because iPSCs can be easily reprogrammed from dermal fibroblasts and then differentiated into hepatocyte-like cells (HLCs), which functionally resemble PHHs (Hu and Li, 2015). iPSC-HLCs can recapitulate metabolic variations observed in the population and have proved to be potent in both short- and long-term drug screening and in investigating hepatotoxicity or developing novel therapeutics (Holmgren et al., 2014; Medine et al., 2013; Szkolnicka et al., 2014). In addition, they have been utilised for studying fetal liver exposure to harmful substances (Lucendo-Villarin et al., 2017) and in identifying noncoding micro-RNAs regulating human liver damage (Szkolnicka et al., 2016; Yang et al., 2016). Furthermore, HLCs have been successfully used in developing *in vitro* models for studying hepatic diseases such as systemic amyloidosis (Leung et al., 2013), liver-stage malaria (Ng et al., 2015) and hepatitis C viral infection (Zhou et al., 2014). iPSC-HLCs could also offer a good model for investigating basic mechanisms of e.g. lipid metabolism as well as its dysregulation related to different diseases such as fatty liver disease or atherosclerosis.

Lipids are a highly diverse class of biological molecules with crucial roles in cellular energy storage (mainly in the form of triacylglycerols), structure (e.g. key components of plasma and nuclear membranes, endoplasmic reticulum and Golgi apparatus, and trafficking vesicles like endosomes and lysosomes) and signalling (as ligands that activate signal transduction pathways as well as mediators of signalling pathways) (Van Meer et al., 2008). Furthermore, *de novo* FA synthesis in the cells has a significant impact on the acquisition and maintenance of cellular pluripotency through increased mitochondrial fission (Wang et al., 2017). Mammalian cells express tens of thousands of different lipid species and use hundreds of proteins to synthesise, metabolise and transport them (Muro et al., 2014). Moreover, lipid defects are central to the pathogenesis of many common diseases such as non-alcoholic fatty liver disease (Ruhanen et al., 2017; Younossi et al., 2016) or atherosclerosis (Meikle et al., 2011; Stübiger et al., 2012). However, because the cellular lipidome is highly complex and very dynamic, the study of lipids and identifying the precise underlying defects has previously been hampered by analytical limitations. This has been resolved by the emergence of advanced lipidomic technologies. Lipidomics aims to precisely define and quantitate the molecular profiles of lipids present in a cell, organism or tissue (Watson, 2006; Wenk, 2005), and provides precise quantitative snapshots of the lipidomes comprising hundreds of different molecules (Llorente et al., 2013; Sales et al., 2016). This technological advancement in conjunction with computational technologies has made this field a promising area for biomedical research (Ekroos, 2012).

In this study, we produced iPSCs from patient-derived dermal fibroblasts and differentiated them into HLCs. In total, we quantified 165 molecular species of lipids, and are the first to report a comprehensive lipidomic profile of cells during the entire differentiation process from iPSCs through the definitive endoderm (DE) stage to HLCs. We assess the occurring lipidomic alterations in relation to the variable supply of different FAs measured in the cell culture media at each stage of the differentiation. Thus, we do not overlook the comprehensive effects of the essential exogenous metabolites the cells acquire from the culture medium. Additionally, we present biochemical and functional measurements and expression of sphingolipid (SL) metabolism-related genes during HLC differentiation.

## RESULTS

### Characterisation of the iPSC lines

The three iPSC lines used in this study – UTA.10100.EURCAs, UTA.11104.EURCAs and UTA.11304.EURCCs – were characterised in detail for their pluripotency. All three iPSC lines expressed characteristic markers of pluripotency at the protein (Fig. S1A) and gene (Fig. S1B) level, and the virally transferred exogenous pluripotency genes were silenced (Fig. S1C). The pluripotency of iPSC lines was proven *in vitro* by embryoid body (EB) formation and performing PCR to show the presence of all three germ layers (Fig. S1D). The karyotypes of all three iPSC lines were normal (Fig. S2).

### Differentiation of iPSCs to HLCs through a DE stage

**Fig. 1.**
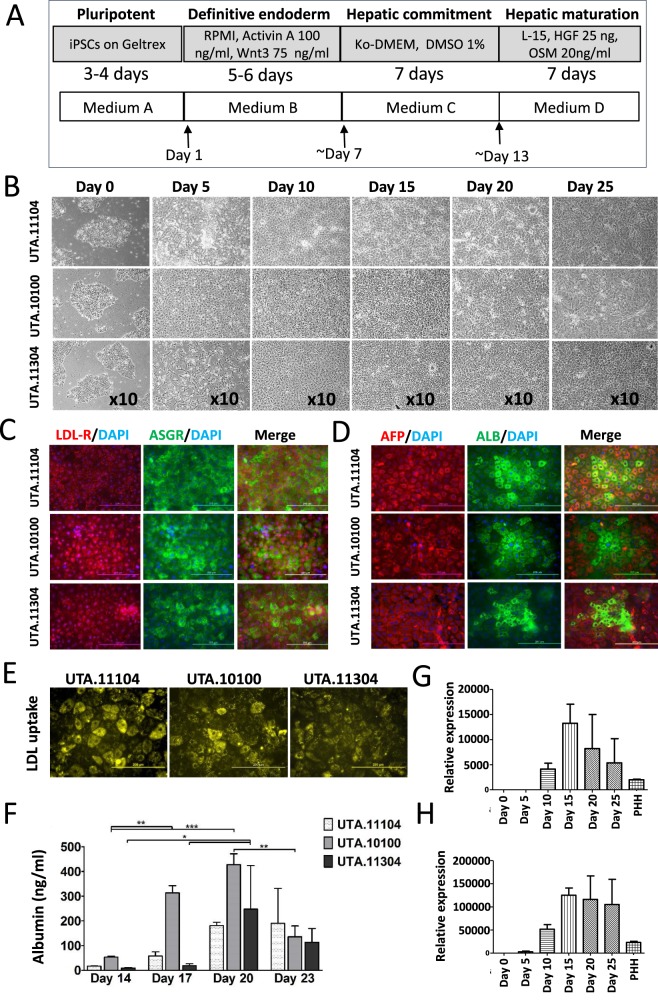
**Hepatic differentiation of iPSCs through** **the** **definitive endoderm (DE) stage.** (A) A schematic representation of the hepatic differentiation protocol (modified from Hay et al., 2008) and cell culture media used at each stage. See text for details of the media. (B) Phase-contrast images showing sequential morphological changes from iPSCs (day 0) to DE (day 5) and finally hepatocyte-like cells (HLCs) (day 20) during differentiation. (C,D) Immunocytochemistry of the iPSC-HLCs differentiated from three patient lines (UTA.10100.EURCAs, UTA.11104.EURCAs and UTA.11304.EURCCs) at day 20 showing the expression of (C) LDL receptor (LDL-R) and asialoglycoprotein receptor (ASGR), as well as (D) α-fetoprotein (AFP) and albumin (ALB). Nuclei are stained with DAPI. (E) The iPSC-HLCs were able to uptake LDL at day 20 of differentiation and (F) produce and secrete albumin during differentiation. Values are normalised per 1 million cells per 24 h. Bars represent means±s.d. of three biological replicates. Gene expression levels of (G) apolipoprotein B (*ApoB*) and (H) apolipoprotein AI (*ApoAI*) at different time points during the iPSC to HLC differentiation normalised to the housekeeping gene *GAPDH*, and expressed relative to day 0. Each sample was run in triplicate and bars represent means±s.d. of three studied cell lines. iPSC, induced pluripotent stem cell; KO-DMEM, KnockOut Dulbecco's modified Eagle medium; DMSO, dimethyl sulfoxide; HGF, hepatocyte growth factor; OSM, oncostatin M; PHH, primary human hepatocyte. Scale bars: 200 μm.

We used a previously described three-step protocol (Hay et al., 2008) to produce HLCs from human iPSCs (Fig. 1A). During the differentiation process, the cells underwent morphological changes: iPSCs gradually lost their typical round and dense morphology and after migration formed spiky-shaped DE cells. During the second week of differentiation, at the hepatoblast stage, cells gradually increased in size and, as they matured during the third week, formed polygonal HLCs with distinct canaliculated borders (Fig. 1B). The immunocytochemical staining at day 5 showed that cells expressed only low levels of pluripotency marker OCT3/4 but were strongly positive for the DE marker SOX17 (Fig. S3A). Furthermore, flow cytometry analyses showed that 71-96% of the cells were positive for the DE marker CXCR4 at day 5 of differentiation (Fig. S3B). These results indicated that the iPSCs had efficiently differentiated into DE during the endodermal induction step of the differentiation protocol.

Consistent with the morphological changes observed during the differentiation process (Fig. 1B), changes in gene expression were detected (Fig. S3C). The expression of *OCT3/4* was clearly downregulated by day 5, whereas the DE marker *SOX17* was highly expressed at this stage. *FOXA2*, a transcription factor involved in liver metabolism (Wolfrum et al., 2004), had a continuous expression pattern starting around day 5 and continuing until the end of differentiation. As the cells differentiated further from DE towards HLCs, they started expressing *AFP* (around day 10, which peaked around day 15-20 depending on the cell line). Expression of *ALB*, the gene for the most abundant liver protein, increased strongly around day 15 and peaked at day 20-25 (Fig. S3C). Immunocytochemical staining further confirmed the expression of hepatic proteins at day 20 of differentiation (Fig. 1C,D). Low-density lipoprotein receptor (LDL-R), asialoglycoprotein receptor (ASGR), alpha fetoprotein (AFP) as well as albumin (ALB) were all expressed in HLCs (Fig. 1C,D). HLCs stained positive for ALB in 13, 18 and 19% of the cells differentiated from UTA.10100, UTA.11104 and UTA.11304, respectively. Both AFP and ASGR stained positive in more than 90% of HLCs in all three cell lines.

### Functionality assessment of mature HLCs

To assess the functionality of the iPSC-HLCs, we performed several experiments. Because liver is the most important organ for LDL catabolism and LDL-R activity, the ability of HLCs to uptake LDL from the culture medium was evaluated. As shown in Fig. 1E, iPSC-HLCs were able to efficiently uptake LDL. The level of secreted albumin was measured from the cell culture medium at days 14, 17, 20 and 23 of differentiation, and all the iPSC-HLCs synthesised and released albumin into the culture medium with maximum secretion at day 20 (Fig. 1F).

Apolipoprotein B (*APOB*) and apolipoprotein A-I (*APOA1*) gene expression levels of the iPSC-HLCs during the differentiation were also measured as a surrogate for estimating very low-density lipoprotein (VLDL) and high-density lipoprotein (HDL) production levels of the cells. APOB is the main protein component of VLDL, and the product of the *APOA1* gene is the main protein component of HDLs. The expressions of both *APOB* and *APOA1* genes rose around day 10, reached their peak at day 15 and then lowered towards the levels of expression in PHHs (Fig. 1G,H). The increasing expression of these liver-specific genes further demonstrated that the iPSCs were differentiating and maturing towards functional hepatocytes.

### Lipidomic profiling of iPSC-HLCs

**Fig. 2.**
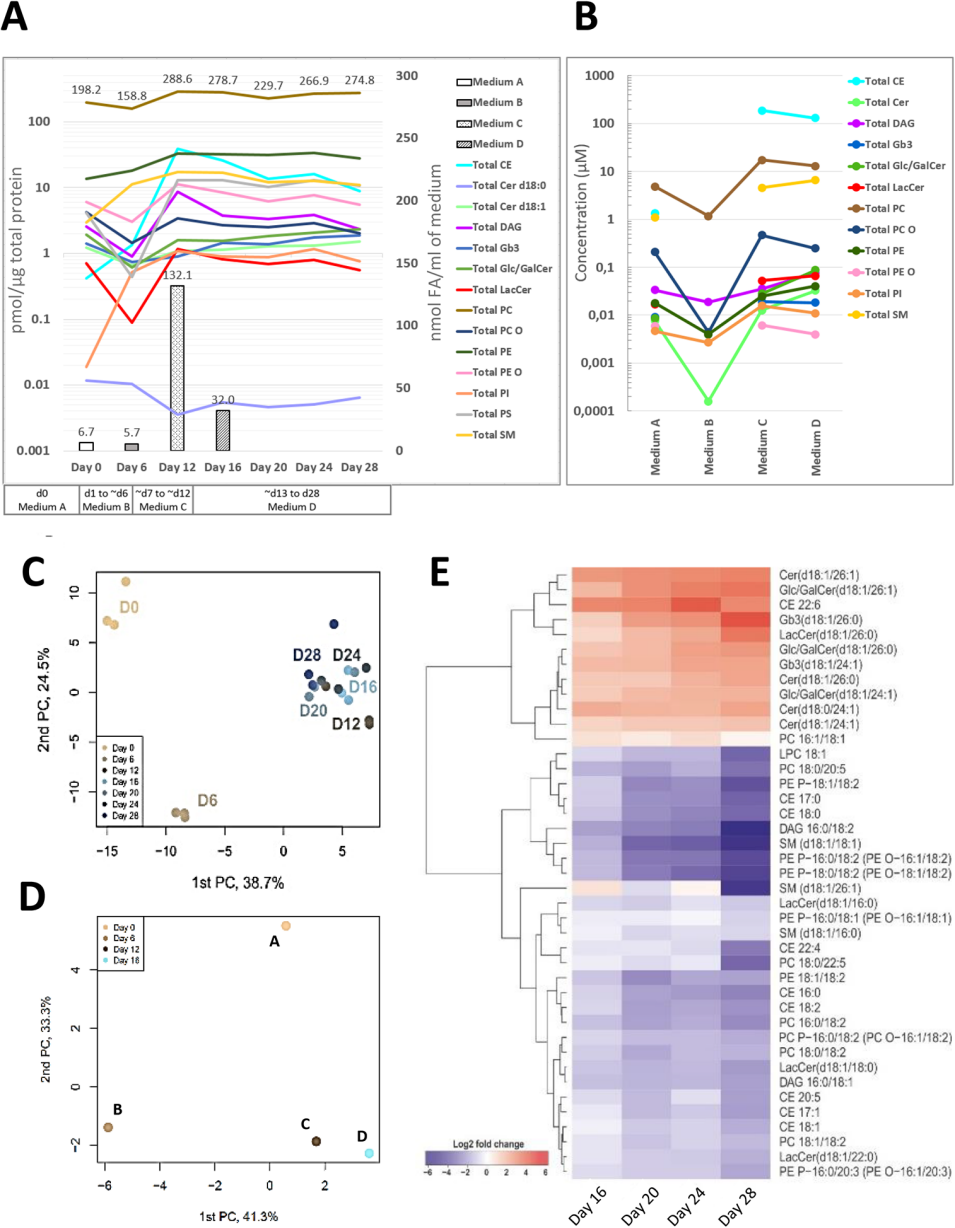
**Changes in the lipidome during iPSC to hepatocyte-like cell (HLC) differentiation.** (A) Lines describe the total concentration of different lipid classes detected in the cells at seven time points during the iPSC to HLC differentiation. Bars represent the total FA concentration (nmol FA/ml of medium) of different media used during hepatic differentiation. Values at the top of bars and the top line show the total concentrations of FAs in the medium and PC in the cells, respectively. (B) Total concentration (µM) of different lipid classes in culture medium A, B, C and D. (C) Principal component analysis (PCA) plot showing the separation of samples based on lipid profiles. PCA analysis of the 165 molecular lipids showed that the two first principal components constitute 63.2% of the variance. (D) PCA analysis of the FA content of cell culture media used at different stages of hepatic differentiation. (E) Lipidomic heat map showing fold increase of molecular lipid species at day 16, 20, 24 and 28 of iPSC to HLC differentiation as compared to day 12. Each horizontal row represents a molecular lipid and each vertical column represents an individual time point of the differentiation. Lipid abundance ratios are coloured according to the fold changes and the colour key indicates the magnitude of log2 fold change. Data shown are the lipids for which concentration differed statistically significantly (*P*<0.05) between day 12 and day 28. iPSC, induced pluripotent stem cell; CE, cholesteryl ester; Cer, ceramide; DAG, diacylglycerol; Gb3, globotriaosylceramide; Glc/GalCer, glucosyl/galactosylceramide; LacCer, lactosylceramide; LPC, lysophosphatidylcholine; LPE, lysophosphatidylethanoamine; PC, phosphatidylcholine; PC O, alkyl-linked phosphatidylcholine; PE, phosphatidylethanolamine; PE O, alkyl-linked phosphatidylethanolamine; PI, phosphatidylinositol; PS, glycerophosphoserine; SM, sphingomyelin.

We performed lipidomic profiling of the cells during the entire differentiation process from iPSCs to HLCs, and observed changes in cellular lipid content and composition. Overall, more than 160 molecular species of lipids including cholesteryl esters (CEs), diacylglycerols (DAGs), phospholipids (PLs) and SLs were detected during the course of differentiation (Table S5). The cellular contents of lipid classes (per protein) were constantly altered during the differentiation. In the beginning, the protein-normalised concentrations of the majority of lipid classes decreased from day 0 to day 6, which mostly mirrors the low supply of lipids or FAs from the culture media at this stage (Fig. 2A,B). However, clear increases were observed in three classes – sphingomyelin (SM), CE and phosphatidylinositol (PI) – independent of the lower or undetectable levels of those lipids in medium B. This was followed by a drastic increase in all lipid classes from day 6 to day 12, which clearly reflected the abundant lipid and FA in medium C (supplied to the cells from ∼day 7 to day 12). Thus, the amount of FAs and lipids available in the media affects the lipid composition and content of the cells. The measured concentrations of medium lipids and FAs were nicely in line with each other (Fig. 2A,B). During the hepatic maturation, from ∼day 12 onwards, changes in the lipid levels were more subtle, in agreement with the constant FA level of medium D, which was given to the cells during the hepatic maturation phase and contained on average 32.0 nmol FA/ml of medium (Fig. 2A). Principal component analysis (PCA) showed that the cellular lipid composition of iPSCs and DE stage cells (days 0 and 6, respectively) clearly differs from the later time points representing hepatoblasts (day 12) and HLCs (day 16-28) (Fig. 2C). A similar PCA plot pattern was detected for the FA composition of cell culture media (Fig. 2D).

To visualise the alterations occurring in the cellular lipidome during the iPSC to HLCs differentiation in more detail, a heat map was computed using day 0 as the reference and including all the detected molecular lipids (Fig. S4A). Because the biggest alterations in the cellular lipid content and composition happened before day 12 as observed in the PCA (Fig. 2C) and appeared mostly to reflect the FA supply from the media, we computed another heat map using day 12 as the reference (Fig. S4B). From this heat map, the lipid remodelling during the hepatocyte maturation phase became more evident as the fluctuation from days 0 to 6 was excluded. We further restricted the heat map to include only molecular lipids, the concentration of which changed statistically significantly between days 12 and 28 (*P*<0.05). In the resulting heat map, 41 molecular lipids divided into two distinct clusters visible on the heat map dendrogram (Fig. 2E). The upper cluster consists of mainly SLs with very-long-chain FAs (C24-26), levels of which increased during the maturation. Lipids that decreased after day 12 are clustered in the lower part of the heat map consisting of several CE species as well as lipids containing FA 18:2 [linoleic acid (LA)]. Similar phenomena were observed in another heat map drawn separately for the 45 FAs present in specific lipid classes in the cells (Fig. S5).

**Fig. 3.**
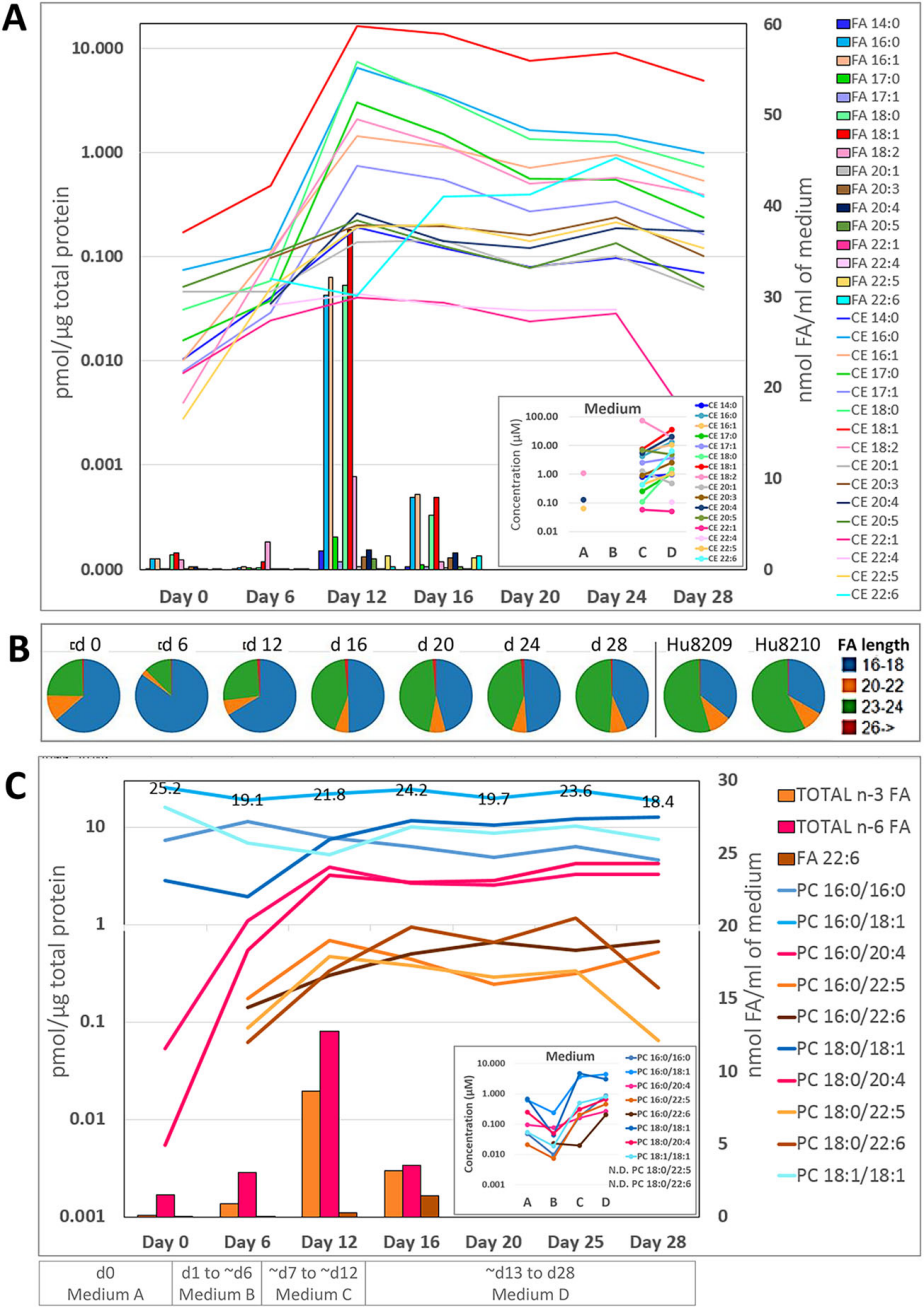
**Changes in cholesteryl ester (CE), polyunsaturated fatty acid (PUFA)-containing phosphatidylcholine (PC) species and the chain length of sphingolipids (SLs) during the iPSC to hepatocyte-like cell (HLC) differentiation.** (A) Lines represent the concentration (pmol/µg total protein) of all detected CE species at different time points of the iPSC to HLC differentiation. Concentrations are presented on a logarithmic scale. Bars represent concentration of all different FAs (nmol FA/ml of medium) detected in different cell culture media used during hepatic differentiation. The insert on the lower right shows the concentration (µM) of CE species detected in culture medium A, B, C and D. No CE species were detected in medium B. (B) The distribution of SLs with different FA chain lengths at different time points shows that, during hepatic differentiation and maturation, the relative abundance of C16-18 species gradually decrease, whereas C23-24 species increase. At the end of the hepatic maturation phase, the distribution closely resembles that of primary human hepatocytes (Hu8209 and Hu8210). All SLs detected were included in the calculations. (C) Lines represent the concentrations (pmol/µg total protein) of selected PC species at different time points. The levels of saturated/monounsaturated FA (in blue)-containing species remain rather constant, whereas levels of PUFA-containing lipid species (20:4 in red; 22:5/22:6 in orange) emerge/increase considerably at day 6 and 12. Bars represent the total *n*-3 FA, *n*-6 FA and FA 22:6 concentration (nmol FA/ml of medium) in cell culture media at each stage of differentiation. The insert on the lower right shows the concentration (µM) of PC species detected in culture medium A, B, C and D. PCs 18:0/22:5 and 18:0/22:6 were not detected in the media. Values at the top line show the concentration of PC 16:0/18:1 in the cells during differentiation.

We detected 16 different CE species, the most abundant during the iPSC to HLC differentiation being CE 18:1, CE 16:0 and CE 18:0 (Fig. 3A, lines), concurring with the higher level of FAs 16:0, 18:0 and 18:1 available in the medium C (Fig. 3A, columns). Interestingly, CE 18:0 concentration was low in medium C whereas CE 18:2 was detected the highest (Fig. 3A, insert). This CE 18:2 loading did not cause comparable accumulation of CE 18:2 in the cells. The majority of CEs peaked at day 12, with the exception of CE 20:1 (peak day 16) and CE 20:3, CE 22:5 and CE22:6 (peak day 24). The decrease of several CEs and the increase of CE 22:6 are clearly visible in the heat maps, illustrating the statistically significant lipid and FA changes between days 12 and 28 (Fig. 2E, Fig. S5). It is of note that only the last medium (medium D, given to the cells from ∼day 13 onwards) provided the cells abundantly with FA 22:6 (Fig. 3A).

During hepatic differentiation and maturation, we detected an overall increase in the FA chain length of SLs (Fig. 3B). The C16-18 species predominated (64%) in the beginning of differentiation but their relative abundance gradually decreased (to 44%) during the hepatocyte maturation process. At day 6, a temporary increase in the proportion of the C16-18 species was detected. Interestingly, as the differentiation progressed, the SL chain length profile approached that of PHHs, in which the very-long-chain species (C20-26) predominate (Fig. 3B).

**Fig. 4.**
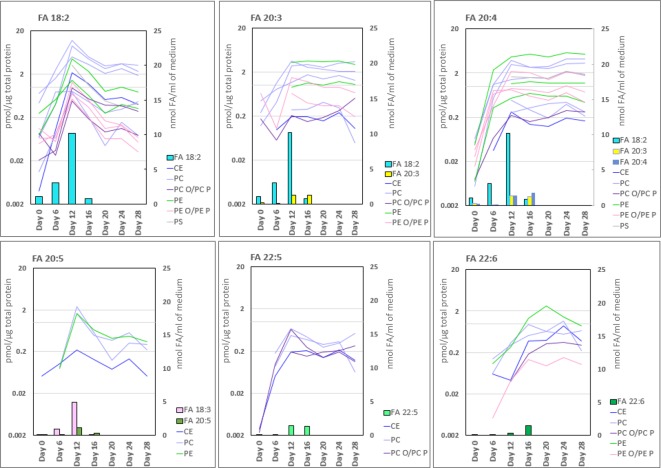
**The levels of polyunsaturated fatty acid (PUFA)-containing lipids during iPSC to HLC differentiation.** Lines in the graphs show concentrations (pmol/µg total protein; logarithmic scale on the left) of lipids containing the given PUFA moiety at different time points. Each line represents the lipid species with the specific PUFA in a particular lipid class; line colour refers to lipid class (colour key depicted on the right). The bars represent the concentration of FA 18:2, 20:3, 20:4, 18:3, 20.5, 22:5 and 22:6 (nmol FA/ml of medium, scale on the right) in the cell culture media during hepatic differentiation. CE, cholesteryl ester; PC, phosphatidylcholine; PC O/PC P, ether-linked PC; PE, phosphatidylethanolamine; PE O/PE P, ether-linked PE; PS, phosphatidylserine; FA, fatty acid.

Closer examination of the PL molecular species revealed that the largest changes occurred in polyunsaturated fatty acid (PUFA)-containing lipid species, whereas lipids comprised of saturated or monounsaturated FAs (SFA or MUFA, respectively) were more constant throughout the entire differentiation (Fig. 3C, Table S5). The polyunsaturated lipid species were essentially absent at the beginning of differentiation and increased drastically until day 12 or 16, reflecting the increasing concentration of *n*-3 and *n*-6 PUFAs in the media during differentiation, as shown in Fig. 3C for the phosphatidylcholines (PCs). On the other hand, the PCs containing FA 22:5 and 22:6 appeared later during the differentiation and at a lower concentration, consistent with their low medium concentration as well as a late and (as compared to *n*-6 PUFAs) low level supply of *n*-3 PUFAs from the medium (Fig. 3C). Lipids with different PUFA constituents mostly peaked around day 12, after which they steadily decreased following the pattern of medium FA 18:2 and 18:3 levels, which were at the highest in medium C (during ∼days 7-13) and then dropped to a lower level for the rest of the differentiation (Fig. 4). The molecular species containing FA 20:3 and FA 20:4 peaked around day 12, reflecting the media rich in their C18 precursors, and then their contents remained at the fairly constant level due to the later moderate supply of FA 20:3 and 20:4 themselves (Fig. 4). The *n*-3 PUFA precursor, FA 18:3, and the successors on the metabolic pathway, FA 20:5 and 22:5, were not able to efficiently raise the levels of the 22:6-containing species, which peaked only during days 16-24, i.e. after switching to 22:6-rich medium (Fig. 4, lower row).

### Gene–lipid interaction

**Fig. 5.**
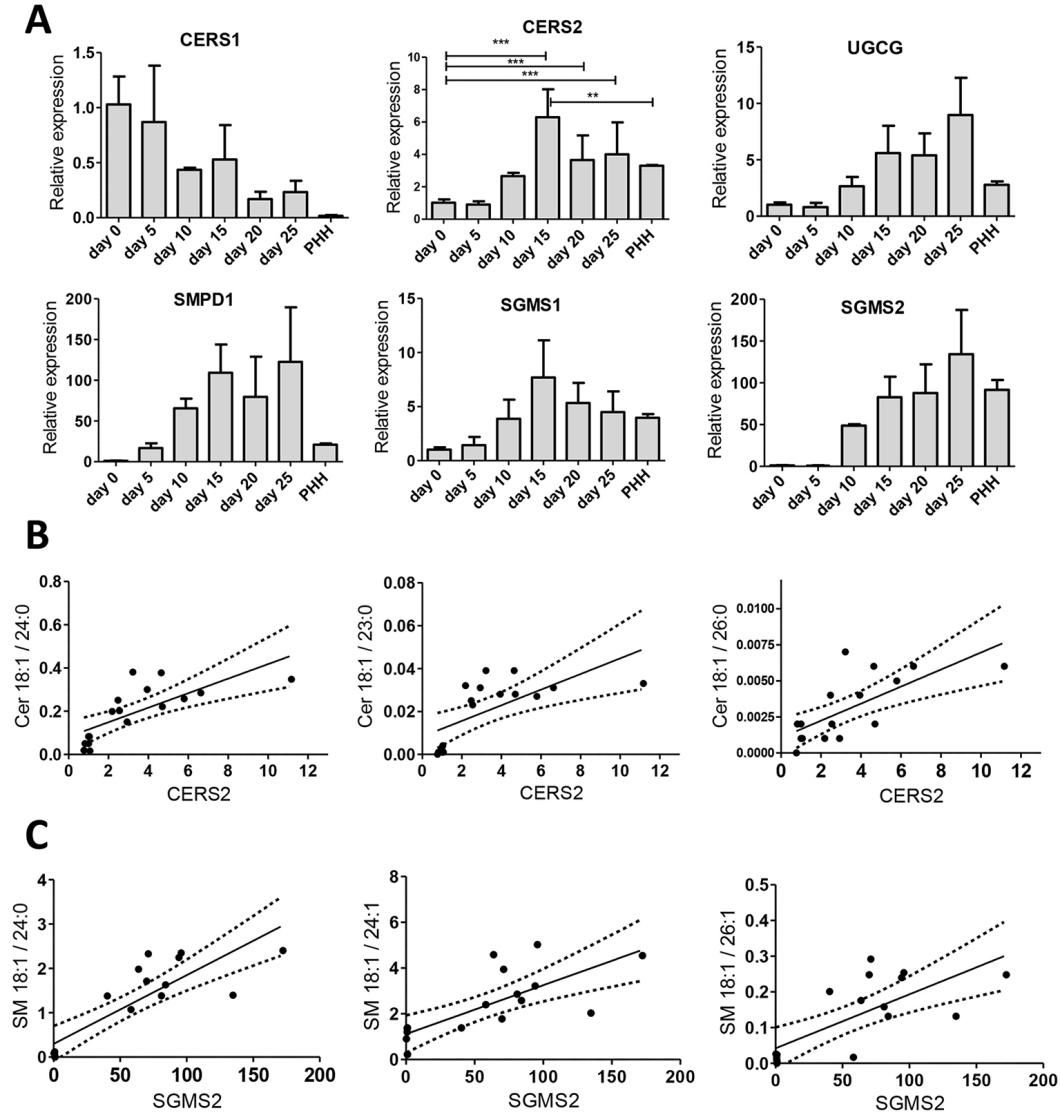
**Expression levels of sphingolipid (SL) metabolism-related genes during hepatic differentiation and in primary human hepatocytes (PHHs).** (A) The expression of *CERS1*, *CERS2*, *UGCG*, *SMPD1*, *SGMS1* and *SGMS2* during hepatic differentiation from iPSCs at day 0, 5, 10, 15, 20 and 25 as well as in PHHs. The expression of each gene was normalised to the endogenous control gene, *GAPDH*, and presented relative to day 0 (iPSCs). Each sample was run in triplicate and bars represent means±s.d. of three studied cell lines or two PHH donors. **P≤0.01; ***P≤0.001. (B) The expression of *CERS2* correlates positively with Cer d18:1/24:0 (Spearman *r*=0.83, *P*<0.0001), Cer d18:1/23:0 (*r*=0.78, *P*<0.001) and Cer d18:1/26:0 (*r*=0.73, *P*<0.001). (C) *SGMS2* expression correlates positively with levels of SM d18:1/24:0 (*r*=0.85, *P*<0.0001), SM d18:1/24:1 (*r*=0.78, *P*<0.001) and d18:1/26:1 (*r*=0.67, *P*<0.01). Cer, ceramide; CERS, ceramide synthase; SGMS, sphingomyelin synthase; SMPD, sphingomyelin phosphodiesterase; UGCG, UDP-glucose ceramide glucosyltransferase; SM, sphingomyelin.

Levels of several ceramides with very-long-chain FAs, e.g. Cer d18:1/26:1 and Cer 18:1/26:0, increased statistically significantly during the hepatic maturation phase (Fig. 2E). Ceramide synthesis involves six different ceramide synthases (CerSs): CerS1-CerS6. The *CERS1* gene was the only highly expressed ceramidase gene at the iPSC stage and it gradually reduced to the expression levels detected in PHHs (Fig. 5A). The expression of the *CERS2* gene, by contrast, slowly increased during hepatocyte differentiation, reaching the expression level in PHHs by day 20 of differentiation (Fig. 5A). A statistically significant correlation was observed between *CERS2* expression and the very-long-chain ceramides Cer d18:1/24:0 (*r*=0.83, *P*<0.0001), Cer d18:1/23:0 (*r*=0.78, *P*<0.001) and Cer d18:1/26:0 (*r*=0.73, *P*<0.001) (Fig. 5B). A similar gene expression pattern to *CERS2* was observed for *CERS3*. *CERS4* expression varied whereas that of *CERS6* gradually increased during the differentiation, and *CERS5* was ubiquitously expressed throughout the differentiation (Fig. S6A).

Ceramides are degraded to sphingosine and free FAs by ceramidases encoded by distinct genes such as *ASAH1* and *ASAH2*. The expression of *ASAH1* increased during the differentiation (Fig. S6B). *ASAH2* expression peaked strongly at day 15 and then gradually reduced towards the levels detected in PHHs. *ASAH2b* expression was constant throughout the differentiation and at the same level as in PHHs, only peaking slightly at day 15 (Fig. S6B).

Lactosylceramide (LacCer) and glucosyl/galactosylceramide (Glc/GalCer) are members of the glycosphingolipid (GSL) family and the UDP-glucose:ceramide glucosyltransferase (*UGCG*) gene encodes the enzyme that catalyses the first glycosylation step in GSL biosynthesis. There was a statistically significant increase in four different GSLs, including LacCer d18:1/26:0 and Glc/GalCer d18:1/26:1, during the hepatic maturation phase (Fig. 2E). Compatibly, *UGCG* expression increased during the iPSC-HLC differentiation (Fig. 5A), and we observed a statistically significant correlation between *UGCG* expression and the total level of Glc/GalCer (*r*=0.54, *P*<0.05), particularly with Glc/GalCer d18:1/26:0 (*r*=0.84, *P*<0.0001) (Fig. S6C).

An almost fourfold increase in total SM level was detected from day 0 to day 28 of the iPSC-HLC differentiation (Fig. 2A). Specifically, an increase in the longer (C23-26) SM species was observed (Fig. S7A). SM is metabolised from ceramides by SM synthases (SMS1 and SMS2). The expression of the corresponding genes, *SGMS1* and *SGMS2*, increased during differentiation, reaching the expression levels of PHHs by day 10 (Fig. 5A). A statistically significant correlation was detected between *SGMS2* expression and levels of very-long-chain SMs (Fig. 5C) such as d18:1/24:0 (*r*=0.82, *P*<0.0001), SM d18:1/24:1 (*r*=0.78, *P*<0.001) and SM d18:1/26:1 (*r*=0.67, *P*<0.01). Levels of these very-long-chain SMs increased independently of the exogenous lipid supplies during the hepatoblast phase, whereas the shorter-chain SMs such as SM d18:1/18:0 and SM d18:1/18:1 decreased at the hepatic maturation stage (Fig. S7A,B). Several different sphingomyelinase (Smase) enzymes degrade SMs. The *SMPD1* gene encodes a lysosomal acid Smase, the expression of which increased during the hepatic differentiation (Fig. 5A) along with the expression of another sphingomyelinase gene, *SMPD3* (Fig. S7C). The expression of two other Smase genes, *SMPD2* and *SMPD4*, was fairly constant through the differentiation and similar to that in PHHs (Fig. S7C).

## DISCUSSION

In this study, we successfully produced functional HLCs from iPSCs and describe a comprehensive lipidomic fingerprint of these cells in conjunction with biochemical and functional measurements. We quantitatively monitored a total of 165 molecular species of lipids during the course of hepatic differentiation, as well as measuring the concentrations of different lipids and FAs in the cell culture media at each stage of differentiation. The analyses of lipids and FAs in the media were complementary in characterising the supplies of the cells. After commitment of the cells to the hepatic lineage (around day 12), protein-normalised concentrations of most studied lipid classes increased. These included functionally important specific PLs and SLs but, as expected, the main building blocks of bulk membrane, PC and PE, showed less variation. At the same time, the levels of storage lipids were elevated, indicated by the increased CE totals. DAGs were also analysed because they are an important intermediary lipid class between the storage and structural lipids, and their elevated levels at day 12 indicate enhanced lipid metabolism (complex triacylglycerol molecular species were not addressed in this study). These lipidome alterations mirror the large increase in medium FA levels and improved supply of PUFAs to the cells. Simultaneously, the cells grew in size and approached hepatocyte cell morphology. Along with the influence of exogenous supplies, we also found endogenous cellular responses: specific SL-metabolism-related genes correlated with distinct SL species levels, which showed that, during the differentiation, changes in gene expression levels were reflected in the levels of specific lipid species at a given time point.

Cholesterol biosynthesis is one of the important functions of hepatic cells. Interestingly, the three most abundant storage forms of CEs detected during the differentiation of iPSCs to HLCs, i.e. CE 18:1, CE 16:0 and CE 18:0, have previously been shown to be among the most abundant CEs in the human liver (Nestel and Couzens, 1966). Despite the level of CE 18:2 being very high in media C and D, when analysing cellular CE levels, this species was not among the highest species but the essential 18:2n-6 [linoleic acid (LA)] was apparently incorporated into structural PLs, priming these PUFAs for signalling. This gives confidence that lipid metabolism of this cell model resembles that of genuine hepatocytes. Most CE species were at their highest levels at day 12 and then, along with decreasing medium CE and FA levels, CEs in the cells decreased during hepatocyte maturation. Compared to medium A and B, medium C (provided to the cells from ∼day 7 to ∼day 13) contained a very high total concentration of FAs, exceeding the sufficient amount for a cell's consumption. This is also consistent with our observation of lipid droplet (LD) formation during the second week of differentiation (results not shown), suggesting that the excess amount of FAs provided in medium C are stored as LDs in the cells. LDs are the main reservoir for neutral lipids in the cells and can be used for metabolism, membrane synthesis (PLs and cholesterol) and steroid synthesis (Martin and Parton, 2006).

SLs form a class of lipids defined by their C18 amino-alcohol backbones, which are synthesised in the ER from non-SL precursors. Modification of this basic structure gives rise to the vast family of SLs such as ceramides, SMs and GSLs, which are structural components of biological membranes and bioactive molecules participating in diverse cellular activities such as cell division, differentiation, gene expression and apoptosis (Gault et al., 2010). SLs also participate in cell signalling and modulate inflammation (Hannun and Obeid, 2008; Maceyka and Spiegel, 2014). Furthermore, increasing evidence shows that SLs contribute to the pathogenesis of metabolic diseases including atherosclerosis (Borodzicz et al., 2015; Haus et al., 2009; Hornemann and Worgall, 2013) and that SL metabolism is affected by dyslipidemia (Holland and Summers, 2008). Because the liver is heavily involved in lipid metabolism, hepatocytes offer a good cell model for studying the basic mechanisms of lipid metabolism and its dysregulation.

The precursor of all SLs is ceramide, which primarily consists of a sphingoid long-chain base (sphingosine) and one FA chain, the length of which typically ranges from C14 to C26, the ceramide species with FAs 16:0, 24:0 and 24:1 being predominant in most mammalian tissues. Ceramide synthesis is a complex process and orchestrated by six mammalian CerSs, each of which produces ceramides with distinct FA chain lengths (Cingolani et al., 2016; Levy and Futerman, 2010; Park et al., 2014). The expression pattern of CerS is cell specific, which is reflected in the different SL acyl chain composition in a given tissue (Levy and Futerman, 2010). CerS2, the dominant CerS isoform found in the liver, utilises C20-C26 acyl-CoA species as substrate and is one of the major CerSs responsible for the synthesis of long-chain ceramide species (C20-C26) (Laviad et al., 2008). In our study, the concentration of several very-long-chain (C22 to C26) SLs increased statistically significantly between days 12 and 28, which indicates an increase in CerS2 activity as the cells matured towards functional HLCs. This was supported by a clear and statistically significant increase of *CERS2* gene expression during iPSC-HLC differentiation. Furthermore, a statistically significant positive correlation between *CERS2* expression and very-long-chain ceramides (e.g. Cer d18:1/24:0) was found. The very-long-chain ceramides produced by CERS2 are essential for liver function (Park et al., 2014); thus, the increase in their levels acts as evidence of hepatocyte functionality. Admittedly, we have not measured CERS2 enzyme activities but only the level of *CERS2* gene expression. Still, both the increased *CERS2* gene expression as well as the increase in long-chain ceramides and other long-chain SLs showed that the stepwise hepatic differentiation protocol produced iPSC-HLCs with a lipid phenotype resembling that of PHHs. Ceramide can be degraded to sphingosine and free FA by ceramidases (Mao and Obeid, 2008). Acid ceramidase (encoded by the *ASAH1* gene) most efficiently hydrolyses ceramide with medium-chain FA components (C12 to C14), whereas the neutral ceramidase (*ASAH2*) prefers long-chain to very-long-chain components (C16 to >C24) (Gault et al., 2010). In the current study, expression of *ASAH1* and *ASAH2b* was rather constant, whereas, concurrent with the increase in very-long-chain ceramide, the expression of *ASAH2* peaked at day 15 and then gradually decreased towards levels in PHHs. These findings suggest that there is a balance between ceramide production by CerS and degradation by ceramidases during differentiation, which could partly explain why ceramide levels stay constant towards the end of the differentiation. The C26 FAs were not detected in any of the culture media and the C26 species of ceramides and SLs (synthesised from their precursor) were still found in the cells, with increasing amounts towards the end of the differentiation process. Thus, the levels and molecular species composition of ceramide and the successor SLs seemed to be endogenously regulated.

Total SM levels increased during iPSC-HLC differentiation. We showed that the corresponding genes for SM synthases – *SGMS1* and *SGMS2* – were expressed in the iPSC-HLCs throughout the differentiation at levels approaching those in PHHs. It has previously been shown that both *SGMS1* and *SGMS2* positively correlate with levels of cellular SM (Li et al., 2007). Our study supports this because the expression of *SGMS1* and *SGMS2* correlated well with several molecular SM species, especially the long-chain ones (C23 to C26). Furthermore, during the DE differentiation stage, a sharp increase in total level of SM was observed, whereas PC and ceramide decreased. SM synthases use ceramide and PC as substrates to produce SM (Liu et al., 2009), offering an explanation for the transient decreases of ceramide and PC in the beginning of the hepatic differentiation. However, this still requires further studies owing to the very complex and dynamic nature of the cellular lipidome, and strong influence of the culture media. Because SM is the most abundant SL in human cells, its coordinated breakdown is an essential part of membrane homeostasis. It occurs by the Smase family and results in the production of ceramide and free phosphocholine. The *SMPD1* gene encodes a lysosomal acid Smase, whereas SMPD2, SMPD3 and SMPD4 are neutral Smases localised to different cellular compartments (Kim et al., 2008). SMPD2 is located in ER, and SMPD4 in both ER and Golgi. The expression of *SMPD1* increased during the hepatic differentiation as early as at DE stage, suggesting that lysosomal degradation of SM might be crucial for differentiation, maturation or functional structure of the HLCs because ceramide, as one of the products of SM catabolism, is considered crucial to the above-mentioned vital cellular processes, as are its derivatives (Delgado et al., 2006; Gault et al., 2010). Pharmacological inhibition of SM *de novo* synthesis decreases not only SM levels but also ceramide and sphingosine (Liu et al., 2009). This clearly emphasises that the metabolic conversions among the SLs in the cell are tightly interconnected. In line with this, we observed a similar gene expression pattern between *SMPD1*, *ASAH1* and *UGCG* during the entire hepatic differentiation.

GSLs are complex carbohydrate-containing SLs and characteristic components of plasma membranes, residing specifically in the membrane microdomains called lipid rafts. GSL metabolism and composition are altered during the proliferation and differentiation of various types of cells (Iwabuchi et al., 2010). LacCer is a precursor in the biosynthesis of complex GSLs, and known to activate a signal transduction pathway leading to cell proliferation. In our study, LacCer d18:1/26:0 as well as three different very-long-chain Glc/GalCer increased during hepatic maturation. There was also a trend of increased expression of *UGCG* (responsible for glycosylation) during the iPSC-HLC differentiation, correlating with the levels of Glc/GalCer. This suggests that active UGCG enzyme function is vital in hepatic maturation because its GSL products are essential players in cell growth, development and differentiation, and mediate cell adhesion and modulate signal transduction (Gault et al., 2010).

We detected an overall increase in the FA chain lengths in the studied SL classes during the differentiation. This might suggest induction of membrane lateral heterogeneity with more lipid rafts incorporating SLs with longer chain lengths as the cells differentiate towards HLCs. The lipid rafts are regarded as small (from 10 to 200 nm) specialised regions of plasma membrane enriched in cholesterol and SLs with very-long and saturated acyl chains. The lipid rafts are more ordered and slightly thicker lipid domains than the surrounding bulk membrane, which contains unsaturated PL molecules. In line with this, we found significant increase in very-long and saturated SLs (24:0 and 26:0) (Fig. S5), as well as high levels of unsaturated PLs provided by medium C from day 7 (Fig. 3C). Signalling and cell-adhesion molecules are localised in lipid rafts, implicating that these domains may form platforms for signal transduction and cell adhesion. Complex SLs are needed in the lipid rafts for example for cell–cell contact and efficient membrane trafficking (Wassall et al., 2004). However, besides cholesterol and SLs, the emergence of the raft domains from bulk membrane also involves unsaturated PLs.

Enrichment of *n*-3 PUFA in the plasma membrane alters the lateral organisation of lipids. Polyunsaturated PLs, especially those with highly unsaturated *n*-3 PUFAs, are sterically incompatible with cholesterol and lipids with long saturated acyl chains, and thus the incorporation of these PUFAs into the membrane PLs forces the raft lipids out of the bulk membrane, inducing raft formation (Wassall and Stillwell, 2009). However, supplementing cells in excess with highly unsaturated FAs, consequently inserted into the membranes, may disturb raft integrity (Turk and Chapkin, 2013). By regulating the sizes and properties of the raft versus non-raft domains, the *n*-3 PUFAs regulate raft-mediated downstream signalling, transcriptional activation and cytokine secretion (Turk and Chapkin, 2013). We detected a strong increase in PUFA-containing PL species during hepatic differentiation. In addition to driving raft formation, the polyunsaturated PLs affect bulk membrane properties, such as fluidity, flexibility and permeability (Rawicz et al., 2008). The changing of these properties affects membrane vesicle formation and thereby lipid and protein trafficking, which needs to be efficient as the cells grow, differentiate and make more membranes (Van Meer et al., 2008). Very recently, Ghini and co-workers showed that supplementing culture medium with 22:6n-3 [docosahexaenoic acid (DHA)] affected both the lipidome and metabolome of HepG2 hepatoma cells. Owing to the supplement, the total contents of cholesterol, SFAs and MUFAs decreased, whereas the PUFA and TAG contents of the cells increased (Ghini et al., 2017). In our study, 22:6n-3 was present at a very low level in media A, B and C, and present mainly in medium D, used during the late hepatic maturation. The precursors of 22:6n-3 (e.g. 18:3n-3), given to the differentiating cells at an earlier phase, did not immediately give rise to 22:6n-3, which is likely owing to the required multistep synthetic pathway. Compared to the synthesis of 20:4n-6 from its precursor 18:2n-6, the metabolism of 22:6n-3 from 18:3n-3 requires two additional chain elongations and a desaturation in the ER followed by a peroxisomal chain-shortening step, the process thus requiring more time and being less efficient than the synthesis of 20:4n-6 (Tigistu-Sahle et al., 2017). The HLCs, however, were able to produce some 22:6n-3 from its precursors, as can be seen from the rising levels of e.g. 22:6-containing PC-P (plasmalogen) during hepatic maturation. Incorporating 22:6n-3 into PS is crucial to activate protein kinase C pathways (Aires et al., 2007; Giorgione et al., 1995) and, apparently, this type of signalling is actively recruited only at late stages of the differentiation.

PC is the major component of eukaryotic cell membranes and the major PL component of all plasma lipoprotein classes (Cole et al., 2012), and is currently the only known PL class to be required for lipoprotein assembly and secretion (Castro-Gómez et al., 2015). PC molecules contain a range of FA chains with varying lengths and double-bond positions (Yamashita et al., 1997). We saw a more drastic increase (at ∼day 12) of PC species containing FA 20:4, the precursor of which is an essential FA (18:2n-6), than PCs containing other PUFAs. This can partly be explained by the concurrent strong rise of *n*-6 PUFAs in the medium. When the cells get enough 18:2n-6 from the medium, they are able to synthesise all the other members of the *n*-6 PUFA family (Russo, 2009). However, cellular remodelling processes prefer producing PC species with 20:4n-6 in the s*n*-2 position of the molecule: such PC species are the preferred substrates for cytosolic phospholipase A2 type IV (PLA2IV), cleaving 20:4n-6 for synthesis of eicosanoids, which modulate immune responses, cell growth and differentiation (Astudillo et al., 2012; Fujishima et al., 1999). Apparently, the capacity of the differentiating cells to produce these lipid mediators arises along with the appearance of 20:4n-6-containing PC species.

It is especially interesting that, in our cells, *APOB* expression peaked and LD were observed at 2 weeks, soon after the maturing HLCs reached their highest levels of 20:4n-6-containing PC. It was recently revealed that defects in proteins such as Lpcat3 and Tm6sf2, needed for efficient incorporation of 20:4n-6 into ER membranes, reduce TAG secretion from hepatic cells (Rong et al., 2015; Ruhanen et al., 2017). Thus, it appears that the 20:4n-6-containing PC species are crucial for the assembly and excretion of TAG-containing mature lipoprotein particles, and are likely needed for the proper function of our cell model, the HLCs. Because these specific PC species regulate both immune functions and lipid metabolism of the cells, it is understandable that, when the levels of these 20:4-containing PCs first increased in the 18:2n-6-rich medium, they did not peak very high (as happened for many 18:2-containing lipids) but remained at a constant level to the end of the experiment, independent of the latest switch of the medium. The levels of 20:4n-6-containing PCs were apparently efficiently regulated to avoid excessive eicosanoid signalling and to adjust the rate of lipid secretion from the cells.

Taken together, we show how the lipidome of stem cells is remodelled in response to supplies available in the cell culture media and as a result of changing lipid-gene expression as the cells differentiate and mature towards functional HLCs. The lipidome and expression of lipid-related genes in HLCs resemble those of the PHHs, and the HLCs display the expected morphology and cellular functions of a functional hepatocyte. The observed elevations in the production of very-long-chain SLs and PUFA-containing PLs during hepatic maturation clearly show that cells efficiently take up FAs from the media, incorporate them and modify simple lipids into more complex ones, which in turn can change the membrane architecture, causing the alterations in cellular functions.

### Conclusions

Here, we show an efficient differentiation of iPSCs to HLCs and demonstrate their functionality determined by their cellular lipidomic fingerprint in conjunction with biochemical and functional measurements and lipid-metabolism-focused gene expression. To our knowledge, this is the first time a lipidomic profile has been acquired of stem cells during the course of their maturation into functional HLCs. We anticipate that the described approach will open up new avenues in lipid-focused stem-cell biology and medicine as it provides detailed maps of the underlying lipid content and metabolism of a cell at a given time point, taking into account the cell culture environment and FA components available in the culture media. This provides a novel tool in utilising the lipidome to follow cell differentiation and maturation and how this is affected under different conditions or stimuli. Thus, we expect that the stem-cell lipidomic fingerprinting through HLC differentiation will facilitate  the production of target cells that more closely resemble the primary human hepatocytes, helping to improve our understanding of many hepatocyte-related diseases and their treatments.

## MATERIALS AND METHODS

### iPSC culture

iPSC lines were maintained at 37°C in 5% CO_2_ on mitotically inactivated mouse embryonic fibroblasts (MEFs; Applied StemCell, cat. no. ASF-1223). Cells were treated with KnockOut Dulbecco's modified Eagle medium (KO-DMEM) supplemented with 20% KnockOut Serum Replacement (Ko-SR), 2 mM GlutaMAX, 0.1 mM 2-mercaptoethanol (2-ME) (all from Gibco), 1% nonessential amino acids (NEAA) and 50 U/ml penicillin/streptomycin (both from LONZA). Medium was supplemented with 4 ng/ml human basic fibroblast growth factor (bFGF; R&D System). To generate HLCs, iPSCs were adapted to feeder-free condition on pre-coated plates with Geltrex (Gibco^®^, 1:100 dilution) in mTeSR medium (=medium A) before hepatic differentiation. Cell lines were checked for mycoplasma contamination.

### iPSC reprogramming

Three iPSC lines (UTA.10100.EURCAs, UTA.11104.EURCAs and UTA.11304.EURCCs) were used in this study. The lines were derived directly from the fibroblast of three individual patients. The study was approved by the Ethical Committee of Pirkanmaa Hospital District (R12123) and written consent was obtained from all fibroblast donors. The collected fibroblasts were induced to pluripotency using the Sendai reprogramming kit (OCT4, SOX2, KLF4, C-MYC; CytoTune; Life Technologies) based on the protocol described by Takahashi et al. (2007). Cell lines were induced according to the manufacturer's instructions and cultured on MEF feeders until characterisation.

### iPSC characterisation

All the iPSC lines were characterised in detail as described before (Manzini et al., 2015). In short, we used PCR to study the expression of endogenous pluripotency genes (*NANOG*, *REX1*, *OCT3/4*, *SOX2* and *c-MYC*) and the absence of virally imported exogenes (*OCT4*, *SOX2*, *c-MYC* and *KLF4*). Immunocytochemistry was performed to study the protein expression of pluripotency markers (Nanog, OCT-3/4, SOX2, SSEA-4, TRA 1-60 and TRA 1-81) (Table S1). The normal karyotype of the iPSC lines was confirmed by performing genome-wide screening for gross chromosomal abnormalities with KaryoLite BoBs (product number 4501–0010, Perkin Elmer) in the Finnish Microarray and Sequencing Centre, as described elsewhere (Lund et al., 2012).

The pluripotency of the iPSCs was verified *in vitro* by the formation of embryoid bodies (EBs) from which RNA was extracted and reverse transcription performed as described previously (Manzini et al., 2015). The expression of marker genes characteristic of endoderm (*SOX17* or *AFP*), mesoderm (*KDR* or *ACTC1*) or ectoderm (nestin or musashi) were studied from EBs by PCR and *GAPDH* was used as an endogenous control. The primer sequences for pluripotency genes and virally imported exogenes as well as for marker genes of the three germ layers are presented in Table S2.

### Hepatic differentiation

Hepatic differentiation was performed according to the protocol developed by Hay et al. (2008). After the colonies became 60-70% confluent, differentiation was initiated by culturing the cells in RPMI1640+GlutaMAX medium (Gibco) supplemented with 100 ng/ml Activin A (R&D Systems), 75 ng/ml Wnt3, 1 mM sodium butyrate (NaB) on the first day and 0.5 mM from day 2, and 2% B27 (Gibco) (=medium B) for 5-6 days to DE stage. Hepatic differentiation was initiated by switching the medium to KO-DMEM+20% Knockout Serum Replacement, 1 mM GlutaMAX, 1% NEAA, 0.1% 2-ME and 1% dimethyl sulfoxide (DMSO) (=medium C) for 7 days. From this point cells were cultured in Leibovitz's L-15 medium (Invitrogen), supplemented with 8.3% fetal bovine serum (FBS) (Biosera), 8.3% tryptose phosphate broth (Sigma-Aldrich), 10 µM hydrocortisone 21-hemisuccinate, 1 mM insulin (both from Sigma-Aldrich), 2 M GlutaMAX, 25 ng/ml hepatocyte growth factor (HGF) and 20 ng/ml oncostatin M (R&D Systems) (=medium D) (Fig. 1A).

### Hepatic characterisation

#### Flow cytometry

DE cells were detached at day 5 by a 7- to 8-min incubation with Gentle Cell Dissociation Reagent (StemCell Technologies) at 37°C and then suspended in 3% FBS before being stained by CXCR4 conjugated antibody (R&D Systems) for 15 min at room temperature (RT). The percentage of CXCR4-positive cells were assessed using a BD Accuri C6 cytometer.

#### Real-time quantitative PCR (RT-qPCR) analyses

RNA samples were collected at days 0, 5, 10, 15, 20 and 25 using the RNeasy kit (Qiagen, cat. no. 74106). cDNAs were generated using the High Capacity cDNA Reverse Transcription kit (Applied Biosystems, USA) according to the manufacturer's instructions in the presence of RNase inhibitor. The PCR reaction was performed by using Power SYBR Green PCR Master Mix (Life Technologies, cat. no. 1408470) and the cDNA was multiplied using the Applied Biosystems 7300 Real-time Sequence Detection system. C_T_ values were determined using 7300 SDS software (Applied Biosystems) and relative quantification was calculated by the 2^−ΔΔCT^ method (Livak and Schmittgen, 2001). The primer sequences are presented in Table S3.

The expression of 18 genes involved in lipoprotein formation or SL metabolism was studied by RT-qPCR with the Biomark HD system (Fluidigm Corp., San Francisco, USA) and using TaqMan assays. cDNA was prepared and pre-amplified according to the manufacturer's instructions, as were the RT-qPCR reactions. TaqMan assays (Life Technologies) used in the RT-qPCR are presented in Table S4. The levels of mRNA expression were normalised to the endogenous control gene, *GAPDH*, and expressed as relative expression compared to the undifferentiated iPSCs (at day 0). Each sample was run in triplicate.

#### Immunocytochemistry

Cultured cells were fixed in 4% paraformaldehyde (PFA) for 20 min at RT. Permeabilization and blocking was performed at the same time by 10% normal donkey serum (NDS), 0.1% Triton-X 100 and 1% bovine serum albumin (BSA) for 45 min. Cells were incubated overnight with primary antibodies (Table S1) in the above solution with reduced NDS to 1%. Antigens were visualised using Alexa Fluor 488- and 568-conjugated secondary antibodies (Table S1). Finally, the cells were mounted with Vectashield (Vector Laboratories Inc., Burlingame, CA, USA) containing 40,6-diamidino-2-phenylindole (DAPI) for the nuclei staining and imaged with an Olympus IX51 phase-contrast microscope equipped with fluorescence optics and an Olympus DP30BW camera (Olympus Corporation, Hamburg, Germany).

#### Functionality of the differentiated iPSC-HLCs

The ability of the iPSC-HLCs to both uptake LDL and secrete albumin was studied using the LDL Uptake Assay kit (Cayman Chemicals, USA) and Human Albumin ELISA Quantitation set (Bethyl Laboratories, USA), respectively, according to the manufacturers' instructions. Secreted albumin was measured from 24-h conditioned medium and normalised to the cell number.

### Lipidomic profiling of cells and FA analyses of cell culture media

#### Lipid sample preparation and extraction

Lipids [CEs, SLs, glycerophospholipids (GPL) and polar glycerolipids] were extracted at days 0, 6, 12, 16, 20, 24 and 28 of differentiation from PBS-resuspended cells by a modified Folch lipid extraction, using chloroform [high-performance liquid chromatography (HPLC) grade], methanol and acetic acid [both liquid-chromatography–mass-spectrometry (LC-MS) grade] for liquid−liquid extraction (Ståhlman et al., 2009), which was performed on 96-well plates employing a Hamilton Microlab Star system (Hamilton Robotics AB, Kista, Sweden). All solvents were purchased from Sigma-Aldrich. Samples were spiked with known amounts of lipid-class-specific non-endogenous synthetic internal standards. After lipid extraction, samples were reconstituted in chloroform:methanol (1:2, v/v) and synthetic external standards were post-extract spiked to the extracts (Heiskanen et al., 2013). Quality control samples (QCs) were prepared along with the actual samples for all lipidomic analyses to monitor the extraction and MS performance. In addition, calibration lines were prepared to determine the linear dynamic range of the MS analyses. QCs and calibration lines were prepared in fresh frozen plasma (FFP) samples (Veripalvelu, Finland).

#### Mass spectrometric analyses and data processing

In shotgun lipidomics, lipid extracts were analysed on a hybrid triple quadrupole/linear ion trap mass spectrometer (QTRAP 5500) equipped with a robotic nanoflow ion source (NanoMate, Advion Biosciences Inc., Ithaca, NJ, USA) as described (Heiskanen et al., 2013). Molecular lipids were analysed in both positive and negative ion modes using either multiple precursor ion or neutral loss scans (Ekroos et al., 2002, 2003). The molecular species of lipids were identified and quantified in absolute [CE; PC; phosphatidylethanolamine (PE); phosphatidylserine (PS); lysophosphatidylcholine (LPC); lysophosphatidylethanoamine (LPE); DAG; SM] or semi-absolute [PI; alkyl-linked phosphatidylcholine (PC-O); alkenyl-linked phosphatidylcholine (PC-P); alkyl-linked PE (PE-O); alkenyl-linked PE (PE-P)] amounts (Ejsing et al., 2006) by normalising to their respective synthetic internal standard and the sample amount. SLs were analysed as described before (Merrill et al., 2005), using an Acquity BEH C18, 2.1×50 mm column with a particle size of 1.7 µm (Waters, Milford, MA, USA). A 25 min gradient using 10 mM ammonium acetate in water with 0.1% formic acid (mobile phase A) and 10 mM ammonium acetate in acetonitrile:2-propanol (4:3, v/v) containing 0.1% formic acid (mobile phase B). SLs were analysed on a hybrid triple quadrupole/linear ion trap mass spectrometer (4000/5500 QTRAP) equipped with an ultra-high-pressure liquid chromatography (UHPLC) system (CTC HTC PAL autosampler and Rheos Allegro pump or Shimadzu Nexera X2) using a multiple reaction monitoring (MRM)-based method in negative ion mode.

Molecular lipids were normalised to the total protein concentration in the cell samples. Total protein concentrations were determined using the Micro BCA Protein Assay kit (Thermo Scientific Pierce Protein Research Products) according to the manufacturer's instructions. Data processing was performed by MultiQuant, LipidProfiler/LipidView (AB Sciex) softwares and SAS.

#### FA analysis

FA composition and concentrations in the cell culture media (medium A, B, C and D, see above for details) used at iPSC stage and at different stages of hepatocyte differentiation were analysed by gas chromatography. First, the lipid residues of nitrogen-dried media were subjected to transmethylation in 1% methanolic H_2_SO_4_ at a temperature of 96°C and under nitrogen atmosphere for 120 min, according to the recommendations of Christie (1993). The FA methyl esters (FAMEs) formed were recovered with hexane and their quantitative analysis was performed using a Shimadzu GC-2010 Plus gas chromatograph with ﬂame-ionization detector (GC-FID). The FAME structures were identiﬁed by Shimadzu GCMSQP2010 Ultra with mass selective detector (GC-MSD). Both GC equipments were equipped with ZB-wax capillary columns (30 m, 0.25 mm ID, 0.25 μm film, Phenomenex, USA). When calculating the composition, the FID responses were corrected according to the theoretical response factors (Ackman, 2007) and, when calculating the concentrations in medium, the FAME 13:0 (not present in the samples) was used as an internal standard. In the Results, the FAs were marked by using the abbreviations: [carbon number]:[number of double bonds] n-[position of the first double bond calculated from the methyl end] (e.g. 22:6n-3).

### Statistical analyses

#### Gene expression data

GraphPad Prism version 5.02 software was used for the data analysis. The standard deviation (s.d.) was calculated from the average of three independent samples. Statistical analyses comparing gene expression levels in more than two groups was performed with one-way ANOVA and *P*<0.05 was considered statistically significant.

#### Lipidomic data

Statistical analyses for lipidomic data was performed with R (version x64 3.2.1). Zero values in the data were imputed with a value corresponding to half of the minimum value of the corresponding lipid across all the samples. PCA was performed for the log2-transformed data applying centering and scaling. Heat maps were generated with gplots package by calculating the mean log2 fold change to samples at day 0 or 12.

#### Correlation analyses

Correlation analyses (Spearman) were performed to study whether the expression of SL metabolism genes pairs with relevant lipid parameters and how strong the potential correlation is.
